# Supplementary material for: The RETurn to work After stroKE (RETAKE) trial: Findings from a mixed-methods process evaluation of the Early Stroke Specialist Vocational Rehabilitation (ESSVR) intervention
Source: PLoS One. 2024 Oct 9;19(10):e0311101. doi: 10.1371/journal.pone.0311101 (PMC11463838; doi:10.1371/journal.pone.0311101)
Supplement: S1 Fig — (DOCX) [file pone.0311101.s001.docx]

**S1 Figure: Core components of the Early Stroke Specialist Vocational Rehabilitation (ESSVR) Intervention**

The ESSVR intervention will be provided by an occupational therapist (OT) with specialist knowledge and skills in stroke rehabilitation and who has received training in using the ESSVR intervention.

The ESSVR trained OT:

1) *intervenes early,*  within 12 weeks of stroke, gives early advice on impact of stroke and on return to work (RTW) to the patient, family members and other healthcare professionals;

2) *assesses the impact of stroke* on the person and their job, and on their family, this includes analysis of work ability, and worksite assessment;

3) *delivers individually tailored vocational rehabilitation* (VR), this includes work preparation, and RTW planning;

4) *communicates openly in writing* with stakeholders about work status;

5) *acts as case coordinator for VR* across all sectors;

6) *provides education, advice & emotional support* to patient, family and employer;

7) *mediates workplace adjustments*, negotiates phased RTW, provides feedback on performance;

8) *monitors RTW to ensure work sustainability*, this includes regular review and employer supported feedback on work performance, progress and modification (where necessary);

9) *explores alternatives* where current work cannot be sustained or is not feasible;

10) *gradual withdrawal of the ESSVR* intervention, the patient can re-access as required;

Occupational therapists providing the ESSVR intervention will be supported by mentors experienced in vocational rehabilitation for the duration of the RETAKE trial.
