# Supplementary material for: The RETurn to work After stroKE (RETAKE) trial: Findings from a mixed-methods process evaluation of the Early Stroke Specialist Vocational Rehabilitation (ESSVR) intervention
Source: PLoS One. 2024 Oct 9;19(10):e0311101. doi: 10.1371/journal.pone.0311101 (PMC11463838; doi:10.1371/journal.pone.0311101)
Supplement: S2 Fig — (DOCX) [file pone.0311101.s002.docx]

**S3 Fig** Assessment of fidelity and factors moderating ESSVR delivery in accordance with the Conceptual Framework for Implementation Fidelity (Adapted from Carroll et al, 2007).
