# Supplementary material for: The RETurn to work After stroKE (RETAKE) trial: Findings from a mixed-methods process evaluation of the Early Stroke Specialist Vocational Rehabilitation (ESSVR) intervention
Source: PLoS One. 2024 Oct 9;19(10):e0311101. doi: 10.1371/journal.pone.0311101 (PMC11463838; doi:10.1371/journal.pone.0311101)
Supplement: S1 File — (PDF) [file pone.0311101.s003.pdf]

RETAKE

FORM 22

ESSVR Intervention – Session Content

Participant Initials		Date of Birth	Day Month Year	Participant ID	Site Code	Trial No

- **To be completed by the therapist for each intervention session delivered – ONE DAY = ONE FORM**
- **Please add the participant identifiers to the header**

Session date (Day Month Year)

Therapist name (First name) (Last name)

Office use only: Therapist ID

Location ☐ Home ☐ Work ☐ Community ☐ Non face-to-face *(e.g. phone, email, online)* ☐ Hospital

If non face-to-face, is this due to COVID-19 restrictions? ☐ Yes ☐ No

	Component	Prompts	Delivered? *(Tick if yes)*	If delivered, time spent
RECOVERY AND WORK PREPARATION	**Early Recovery Assessment**	*Medical history, social situation, pre-morbid lifestyle, job/workplace assessment*	☐	mins
	**Current Issues**	*Medical, social, financial, family*	☐	mins
	**Goals** *(Plan / review / monitor)*	*SMART goals, COPM*	☐	mins
	**Homework** *(Tasks set / reviewed)*	*Symptom management, work preparation*	☐	mins
	**Family / Carer Support**	*Stroke education, relationships, adjustment, local resources*	☐	mins
	**Physical**	*Pain, vision, upper limb, communication, sensory, assistive devices, adaptations*	☐	mins
	**PADL / IADL**	*Dressing, eating, meals, budgeting, shopping, writing, use of phone*	☐	mins
	**Mobility**	*Walking, using public transport, road safety, route finding, driving*	☐	mins
	**Cognition**	*Perception, executive skills, memory aids / strategies, problem solving*	☐	mins
	**Psychological Issues**	*Confidence building, mood, anxiety, adjustment, relationships*	☐	mins
	**Fatigue Management**	*Education re: physical and cognitive fatigue, fatigue management strategies e.g. pacing*	☐	mins
	**Work Preparation**	*Skills for work, work simulation, discussing work options, participant keeping contact with employer*	☐	mins
GRADED RETURN TO WORK (RTW)	**RTW without direct employer contact**	*Discussing RTW strategies, providing written RTW plans*	☐	mins
	**RTW with direct employer contact** ↓	*Planning & RTW meeting, grading RTW, review meeting with manager / HR / OH*	☐	mins
	Please specify method of contact with employer	☐ Telephone call ☐ Face-to-face ☐ E-mail ☐ Other, please specify ____		
	If non face-to-face, is this due to COVID-19 restrictions? ☐ Yes ☐ No			
JOB RETENTION / NO RTW	**Monitoring Job Retention**	*Feedback about performance, discussion about intervention ceasing*	☐	mins
	**Job Redirection**	*Activities to support participant into job seeking, discussion about intervention ceasing*	☐	mins

Form continues >>

Completed by ____ Date (Day Month Year)

Form continues on next page ►►

Prior to returning this form to CTRU you must make a copy of the form and any amendments for retention at site. CTRU, University of Leeds (please see Investigator Site File for full contact details).

For office use only	Computerised	Verified/Checked
	Date Initials	Date Initials

ISRCTN12464275

RETAKE

ESSVR Intervention – Session Content

Participant Initials		Date of Birth	Day / Month / Year	Participant ID	Site Code	Trial No

Status at End of Session

Has the participant returned to work? ☐ Yes ☐ No

If yes, date returned to work: Day / Month / Year

Days per week

Hours per day

If no, please specify

- ☐ Intends to return to work
- ☐ Intends to return to work but currently furloughed due to COVID-19
- ☐ Participant has been made redundant due to COVID-19
- ☐ Employer is currently closed due to COVID-19
- ☐ No longer intends to return to work, please give details

Has the participant been discharged from ESSVR intervention? ☐ Yes ☐ No

If yes, please update **F21 Participant Summary** or **F24 Re-engagement** if not the first episode of care

Therapist Time

Travel time *(return journey)* ___ mins

or tick if ☐ N/A

Please summarise any other activity completed:

or tick if ☐ No further activity on this date

**Activity** *(Tick all that apply)*	**Method of activity** *(Tick all that apply)*	**Who was involved?** *(Tick all that apply)*	**Duration**
☐ Administrative	☐ Clinical notes	☐ Therapist	___ mins
☐ Liaison	☐ Letter	☐ Participant (non- clinical contact)	
☐ Session preparation	☐ Telephone call	☐ Relative/friend/carer	
☐ Other, specify	☐ E-mail	☐ Employer	
	☐ Text	☐ Health & social care service(s)	
	☐ Other, specify	☐ Other, specify	

Completed by ______ Date Day / Month / Year

Last Page ■

Prior to returning this form to CTRU you must make a copy of the form and any amendments for retention at site. CTRU, University of Leeds (please see Investigator Site File for full contact details).

**For office use only**	Computerised		Verified/Checked	
	Date	*Initials*	*Date*	*Initials*
