# Supplementary material for: The RETurn to work After stroKE (RETAKE) trial: Findings from a mixed-methods process evaluation of the Early Stroke Specialist Vocational Rehabilitation (ESSVR) intervention
Source: PLoS One. 2024 Oct 9;19(10):e0311101. doi: 10.1371/journal.pone.0311101 (PMC11463838; doi:10.1371/journal.pone.0311101)
Supplement: S2 File — (DOCX) [file pone.0311101.s004.docx]

**S6 File: Interview Topic Guides**

**Stroke Survivor**

**Opening/Introduction:** Thanks for participating in the study and for giving up this time.

**Reminder of the focus of the study:** We are interested in finding out about your stroke, the care you received and any support you received to return to work.

1. Tell me a little about when your stroke happened and how you have been doing since your stroke.

- *Where were you/what were you doing when you had your stroke?*
- *How did you find out you had had a stroke?*
- *What happened in hospital?*
- *How did your stroke affect you? (physically, emotionally, socially, family life, etc)*

1. Tell me about your work situation before you had the stroke.
   - - *Part-time? Full-time? Voluntary? Redundancy?*
     - *Responsibilities/Job role*
     - *Relationships (good relationship with employer?)*
2. Tell me about the treatment and support you’ve received since your stroke in relation to you returning to work (if any)
   - *Who provided it?*
   - *When did they start working with you?*
   - *What aspects of your recovery do you feel they took into consideration?*
   - *What kind of things did you do as part of your treatment?*
   - *How long were you supported for?*
   - *Did they discuss returning to work with you?*

- *Overall, what did you think about the treatment and support you received?*
- *Was there any other help you would have liked to have received?*

1. *Have you or have you not returned to work?* ***If yes:*** *How long have you been back at work?*
   - *Phased return, part time, full time? Paid? Voluntary?*
   - *Can you tell me about your return to work process?* ***OR*** *Can you tell me about why you haven’t returned to work?*
   - ***If back at work:*** *How have you found your return to work? What was helpful or unhelpful in the return to work process?*
   - ***If back at work:*** *How has work changed since your stroke? Have any adjustments or adaptations been put in place? (e.g., changes to role/responsibilities, use of specialist equipment, use of energy conservation techniques, changes in how they travel to work?)*
   - ***If back at work:*** *What kind of support, if any, have you received since returning to work?*
     - *From OT*
     - *From employer*
     - *From family*
     - *Anyone else?*
2. ***Has anything got in the way or made it difficult for you to return to work?***
3. Was returning or not returning to work the right decision for you?
4. What are your plans for the future? (If not back at work - does this include going back to work?
5. What role do you think the occupational therapist (or other health professional) played in your return to work (or non-return to work)?

- *Did they provide any information, advice, or make any suggestions?*
  - *If so, was this helpful?*
  - *Was there anything that could have been improved?*

1. Did you discuss your return with anyone in your workplace (e.g., line manager, HR)?
   - *What happened when you discussed it with them?*
   - *Were they supportive, knowledgeable about stroke?*
2. Would you recommend the support you received to other people?
3. Do you think more people should have access to the same treatment and support you’ve received?
4. Now I want to ask you some questions about the occupational therapist/health professional who helped you return to work.
   - *Can you tell me about your relationship with your OT?*
     - *How did you work together?*
     - *How did you communicate (via phone/email?). How did this work for you?*
     - *What things about your OT helped or did not help your relationship, or your return to work?*
     - *Is there anything you would change about your OT or your relationship with your OT?*
   - *Do you think you and your OT agreed on your return to work goals? Who set the goals?*
   - *Who (between you or your OT) do you think led your return to work?*
   - *Was this an effective relationship for you?*
   - *Did you agree with the OTs advice and suggestions?*
     - *Is there anything you agreed or disagreed with the OT on?*
     - *Did you find yourself taking their advice and suggestions?*

Is there anything else you would like to add or comment on regarding:

- The treatment and support you received in getting back to work?
- Your participation in the study
- Your OT/health professional

**Semi-Structured Interview with OTs following ESSVR Training**

Thank you for participating in the study and for giving up this time, we value your contribution. Today I am interested in finding out your experience of the training and mentoring you received and your experience of implementing ESSVR in practice.

| Questions |
| --- |
| **Background/Experience**  Can you start by telling me about your background as an OT?  *What training have you had as a stroke specific OT?*  *What training and experience have you had of VR?*  *How long have you worked for this NHS Trust? And what roles have you worked in?*  Before the trial, what experience of research have you had (what was your dissertation for your OT qualification), have you done anything else like this?  *What experience have you had of working on another research trial?* |
| **VR in existing service**  Thinking about the service that you currently work in, did it or does it now offer any vocational rehabilitation?  Can you describe what is offered, to whom and when?  What do you know of *any services/voluntary organisations that provide VR?*  *Tell me about the VR that they provide (referral criteria, diagnosis seen, who sees the person (OT, Physio, volunteer), what do they actually do etc)*  How similar and/or different is RETAKE to what your usual OT role?  *What is new to you?*  *What is new to your service?* |
| **Introducing RETAKE**  How did you become involved with the RETAKE trial?  *Did you want to be involved?*  *What made you want to get involved?*  *How did your feelings about the trial change over time?*  *If you knew what you know now at the start would you still have taken part in RETAKE?* |
| **Training**  What did you expect to get out of the training package?  Tell me about the training sessions you attended (initial 2-day + refresher)  *What was useful about the training?*  *Was there anything that was definitely poor? If so what?*  *What could have been improved?*  Did the training (which includes the 2-day teaching, the manual and mentoring) prepare you and support you to deliver the intervention?  How did the training prepare you to deliver the intervention?  *Was there any additional information that you would have liked?*  How could the training be adapted for future therapists?  In this trial we have been testing the RETAKE OTs’ competence to deliver this intervention.  Why do you think the trial wanted to test therapist competence?  Did we do this in the right way?  If yes, what was appropriate?  If no, what would you do differently?  Of the training package (initial training, refresher, manual and mentoring) what was the most important/useful?  *If you could have designed the training/intervention how would you have done it?* |
| **Mentoring**  So thinking about the mentoring you received in more detail.  Did the mentoring help support you to deliver the research?  *If yes, how?*  *If no, why not? What would you have wanted from your mentor?*  What do you perceive as the role of the mentor?  *Could you have managed without your mentor/peer group?*  *How have they supported you to utilise the training you have received?*  *How often do you have contact with your mentor? (not just teleconferences, but also email, calls, texts etc)*  *Do you think you received sufficient support from your mentor?*  *What do you think about the group format of mentoring?*  *How would you improve the on-going support?*  *If the training and intervention were rolled out in the NHS, how do you think this could be accommodated?*  Would you recommend future trials to incorporate mentoring into their trials?  *If so, in what format?*  *By whom?* |
| **Site file**  You also received a Therapy site file and in it was the manual about the intervention. Where have you kept the file?  How often have you referred to the actual intervention manual (you may need to describe it to the OT even…)?  How useful has the manual been?  Can you give me an example of when it has or has not been useful?  As part of the manual, you were also sent electronic templates of letters and reports. Did you manage to put your NHS Trust logo onto these and use them?  How did you change the templates to suit yourself/your style/needs better?  What else have you requested from your mentor or the Nottingham RETAKE team in terms of templates, example letters etc?  At the refresher training, you might have shared some of your own communication and seen others’. How useful was this?  How could improvements be made to help other OTs with the written communication part of the intervention? |
| **Delivering ESSVR**  How does your RETAKE role differ to your usual care role?  How many participants have you seen to date?  So now that you have seen some participants, How challenging does it feel? In what ways?  When you deliver the intervention how familiar does it feel?  How confident do you feel delivering the intervention?  *Can you give me an example of how your confidence has developed?*  What have been some of the “firsts” for you e.g. 1^st^ workplace visit, 1^st^ interaction with an employer etc.?  What have been some of the barriers/difficulties/challenges?  What has helped you to deliver the intervention on a day-to-day basis?  What do you feel is your biggest success?  Is there anything you wish you had done differently?  There were 2 RETAKE OTs and it was anticipated you could work as a team to deliver the intervention. How much do you feel you were in a team? E.g. with the other OT, site recruiters.  What relationships did you have with the wider RETAKE team? From Nottingham? From the Trials Unit in Leeds?  Given that RETAKE is different way of working, how do management support your involvement in the RETAKE trial?  How supported have you felt from the rest of colleagues? i.e. when you have not been able to see a usual care participant for instance, when you have had to have a higher caseload than usual etc.?  How have you been paid/compensated for the time you have given to RETAKE? (Extra hours pay, TOIL, backfill etc.)  What changes have you had to make personally to deliver the intervention? (worked extra hours, different days, had to change home arrangements e.g. childcare) i.e. what has been the personal burden for the RETAKE OT? |
| What do you think are the benefits of the intervention for helping stroke survivors to RTW?  *What do you think is the most valuable part of the intervention? (e.g. early intervention, liaison with employer)*  *Do you think your participants benefitted from the intervention?*  Can you identify any negative aspects for stroke survivors?  Could this intervention be rolled out in your NHS Trust? Can you explain your answer?  *If so, how would you see it working?*  Where do you see the experience of RETAKE taking you in the future? |
| Is there anything else you would like to tell me? |

Tell the interviewee what happens next with their recording etc.

| **Therapist Interview Topic Guide: Treating OT Intervention participants**  Opening/introduction:  Thanks for participating in the study and for giving up this time.  Reminder of the focus of study |
| --- |
| 1. Can you describe the aim of the intervention? 2. Can you describe the mechanism of the intervention – how was it supposed to work? 3. Did this make it different to usual care, and if so how? 4. Was there any part of the intervention that you consider was unnecessary? If yes, why? 5. Was there any further content to the intervention that you think should be included? If yes, why? |
| 1. Do you think this intervention was useful? 2. Did you find the training (manual, teaching, mentoring) useful; and did it equip you to deliver the intervention? 3. Was taking part in the training a worthwhile investment of your time? |
| 1. How compatible is the intervention with the existing stroke care pathway? 2. In relation to any other local RTW services, did this intervention complement those services, duplicate them or fill a gap? 3. Can you describe how managers, colleagues and external organisations (including employer) supported you? |
| 1. Can you tell me about your experience of delivering this intervention overall?  - time/resources invested in delivery vs impact - barriers to delivering the intervention; and how to address barriers?   14. Were you able to see that there were any benefits or dis-benefits, and what were these?  15. Did you learn anything about what would be needed to roll out the intervention effectively?   - changes to intervention; changes in services/resources needed for delivery) |
| 16. Can you tell me about [CASE STUDY PARTICIPANT]?   - What was your relationship like? - How did you work together? - How did you communicate (via phone, email etc)?   - How did this work for you? - Is there anything you would change about your relationship with [CASE STUDY PARTICIPANT]?   17. Tell me about [CASE STUDY PARTICIPANT]’s engagement with you and the intervention?   - Do you feel like they listened to your advice or took your suggestions?   18. Do you feel that you and [CASE STUDY PARTICIPANT] agreed on the goals set and the treatment plan?   - Who set the goals?   19. What about the participant particularly helped or hindered their return to work? (Individual level – i.e. attitudes and behaviours)  20. What other factors about [CASE STUDY PARTICIPANT] helped or hindered their return to work? (On a more general scale – family/employer/environment)  21. Do you feel that you were able to deliver the whole intervention with [CASE STUDY PARTICIPANT]?   - If not what aspects were not delivered and why? - Did you have to adapt the intervention in any way for this participant? If yes, please explain.   22. Do you think the intervention was appropriate for the participant? Why or why not?  23. What further support (if any) do you think would have benefitted [CASE STUDY PARTICIPANT]? |
| 24. Is there anything else you would like to add or comment on regarding:   - The intervention - Your experience of delivering the intervention - [CASE STUDY PARTICIPANT] |
