# Supplementary material for: The RETurn to work After stroKE (RETAKE) trial: Findings from a mixed-methods process evaluation of the Early Stroke Specialist Vocational Rehabilitation (ESSVR) intervention
Source: PLoS One. 2024 Oct 9;19(10):e0311101. doi: 10.1371/journal.pone.0311101 (PMC11463838; doi:10.1371/journal.pone.0311101)
Supplement: S1 Table — (DOCX) [file pone.0311101.s005.docx]

**S2 Table Normalisation Process Theory Constructs and Components**

| **NPT constructs** | **Components** | **Description** |
| --- | --- | --- |
| **Coherence** | - Differentiation - Communal specification - Individual specification - Internalisation | This relates to the “sense making” that people do individually and collectively when faced with implementing changes to existing working practices. |
| **Cognitive Participation** | - Initiation - Enrolment - Legitimation - Activation | This describes the work that people need to do to engage with and commit to a new set of working practices. It also relates to the way people work together to implement new ways of working in a sustainable manner. |
| **Collective Action** | - Interactional workability - Relational integration - Skill set workability - Contextual integration | This explores the work that will be required of people to implement changes in practices. This may require rethinking how existing work practices and the division of labour in a setting will have to be changed in order to be able to implement the new practices. It also considers the skills that are needed to be able to implement new ways of working. |
| **Reflexive monitoring** | - Systematisation - Communal appraisal - Individual appraisal - Reconfiguration | This is the on-going informal and formal appraisal of the effectiveness of changes in working practices – both from an individual and collective perspective. It also considers the potential benefits of the new way of working. |
