# Supplementary material for: The RETurn to work After stroKE (RETAKE) trial: Findings from a mixed-methods process evaluation of the Early Stroke Specialist Vocational Rehabilitation (ESSVR) intervention
Source: PLoS One. 2024 Oct 9;19(10):e0311101. doi: 10.1371/journal.pone.0311101 (PMC11463838; doi:10.1371/journal.pone.0311101)
Supplement: S3 Table — (DOCX) [file pone.0311101.s007.docx]

**S7 Table: Competency Assessment Rubric: Occupational Therapists**

| Criteria | Needs support | Competent | Highly competent |
| --- | --- | --- | --- |
|  | ≤49%  Demonstrates some understanding of ESSVR and its application in RETAKE. However, major deficits noted in VR knowledge, clinical reasoning and application. Requires additional individualised mentoring until next assessment. | 50-69%  Understands ESSVR with some evidence of misinterpretation in its application in RETAKE. Ad hoc monitoring via group mentoring until next assessment. | ≥70%  Fully understands ESSVR and its application in RETAKE. |
| Knowledge of intervention processes, timeframes & documentation  (40% of total marks) | Most answers were missing the required ESSVR components. | Some answers were missing the required ESSVR components. | Few, if any of the required ESSVR components were missing in the answers. |
| Clinical reasoning – identification and analysis of salient work-related issues in the case study, to inform the design of an appropriate intervention (ESSVR) plan in the letter/report.  (50% of total marks) | Limited identification of and/or limited analysis of work-related issues from the case study. None or few solutions for the work-related issues identified within the intervention plan(s). Significant gaps remain in problem-solving. | Some identification of and/or some analysis of work-related issues from the case study. A number of solutions for the work-related issues identified within the intervention plan(s) but a few gaps remain in problem-solving. | Identification and or analysis of all work-related issues from the case study. Comprehensive solutions for the work-related issues within the intervention plan(s). |
| Written communication of work issues. Appropriate use of lay language in letter/report to ensure fitness for purpose & likelhood of engaging reader.  (10% of total marks) | Letter/report lacks logical structure. Limited focus of work issue(s) addressed. Overuse of medical terminology. Little use of lay language to communicate issues. Information conveyed in a manner less likely to engage recipient. | Case study letter/report reasonably well structured. Mostly focussed on the work issue(s) being addressed. Minimal use of medical terminology. Good use of lay language to communicate issues. Information conveyed in a manner may to engage recipient. | Case study letter/report very well structured. Report fully focussed on work issue(s) addressed. Issues communicated clearly in lay language and without any use of medical terminology. Information conveyed in a manner likely to engage recipient. |
