# Supplementary material for: The RETurn to work After stroKE (RETAKE) trial: Findings from a mixed-methods process evaluation of the Early Stroke Specialist Vocational Rehabilitation (ESSVR) intervention
Source: PLoS One. 2024 Oct 9;19(10):e0311101. doi: 10.1371/journal.pone.0311101 (PMC11463838; doi:10.1371/journal.pone.0311101)
Supplement: S4 Table — (DOCX) [file pone.0311101.s008.docx]

**S8 Table: Participating sites**

| **Site** | **Trust Designation** | **NHS Region** | **Number of Intervention arm participants** | **Number of OTs^1^** |
| --- | --- | --- | --- | --- |
| 1 | Acute and Community | North East and Yorkshire | 33 | 4 |
| 2 | Acute and Community | North West | 8 | 3 |
| 3 | Acute and Community | Midlands | 29 | 5 |
| 4 | Acute and Community | South West | 30 | 3 |
| 5 | Community | Midlands | 19 | 4 |
| 6 | Community | London | 7 | 5 |
| 7 | Acute and Community | Wales | 16 | 2 |
| 8 | Acute and Community | London | 31 | 4 |
| 9 | Acute and Community | South West | 17 | 4 |
| 10 | Acute and Community | South East | 8 | 2 |
| 11 | Acute and Community | North East and Yorkshire | 19 | 3 |
| 12 | Acute and Community | South East | 17 | 2 |
| 13 | Community | East | 26 | 3 |
| 14 | Acute and Community | London | 20 | 3 |
| 15 | Community | London | 22 | 2 |
| 16 | Acute | Midlands | 22 | 2 |
| **TOTALS** |  |  | **324** | **51** |
