# Supplementary material for: The RETurn to work After stroKE (RETAKE) trial: Findings from a mixed-methods process evaluation of the Early Stroke Specialist Vocational Rehabilitation (ESSVR) intervention
Source: PLoS One. 2024 Oct 9;19(10):e0311101. doi: 10.1371/journal.pone.0311101 (PMC11463838; doi:10.1371/journal.pone.0311101)
Supplement: S5 Table — (DOCX) [file pone.0311101.s009.docx]

**S9 Table: Demographic data Case-study and additional interview participants (stroke survivors):**

|  | Case Studies (n=26) | | | Additional interview (n=18) | | |
| --- | --- | --- | --- | --- | --- | --- |
|  | UC-Only | ESSVR | Total | UC-Only | ESSVR | Total |
| Gender: %  *Female*  *Male* | 27%  73% | 13%  87% | 19%  81% | 20%  80% | 13%  88% | 17%  83% |
| Age in Years  Mean (SD) | 59.6 (11.7) | 53.3 (8.2) | 56 (10.1) | 54 (9.4) | 61 (8.2) | 57 (9.2) |
| Self-Employed: % | 36.3% | 20.0% | 27% | 20% | 25% | 22.2% |
| Months elapsed between recruitment and interview  *T1*  *T2*  *T3*  *Carer*  *OT* | 3.3(2.6)  8.3(1.5)  12.9(1.4)  12.5(12)  - | 4.6(1.2)  8.1(1.8)  12.5(1.5)  8.7(5.3)  14.2(6.6) | 3.9(2.3)  8.2(1.6)  12.7(1.4)  9.8(6.8)  - | 14 (4.8)  -  -  -  - | 15.75(7.3)  -  -  -  - | 14.8(5.9)  -  -  -  - |
