# Supplementary material for: The RETurn to work After stroKE (RETAKE) trial: Findings from a mixed-methods process evaluation of the Early Stroke Specialist Vocational Rehabilitation (ESSVR) intervention
Source: PLoS One. 2024 Oct 9;19(10):e0311101. doi: 10.1371/journal.pone.0311101 (PMC11463838; doi:10.1371/journal.pone.0311101)
Supplement: S6 Table — (DOCX) [file pone.0311101.s010.docx]

**S10 Table** **Stroke severity measured using OCS and EQ5D mobility question (case-study participants)**

|  | **ESSVR intervention (plus usual care) (n=15)** | **Usual care only (n=11)** | **Total (n=26)** |
| --- | --- | --- | --- |
| **Number of Impairments** |  |  |  |
| None | 80.0% | 36.4% | 61.5% |
| At least one | 20.0% | 63.6% | 38.5% |
| *1* | 13.3% | 63.6% | 34.6% |
| *2* | 6.7% | 0% | 3.8% |
| *3* | 0% | 0% | 0% |
| **Type impairment (not mutually exclusive)** |  |  |  |
| Mobility (EQ5D Mobility Score^^[[1]](#footnote-1)^^)? | 13.3% | 54.5% | 30.8% |
| Aphasia (OCS Picture Naming Score^^[[2]](#footnote-2)^^)? | 13.3% | 9.1% | 11.5% |
| Cognitive (OCS Mixed Score^^[[3]](#footnote-3)^^)? | 0% | 0% | 0% |
| **Type of impairment (mutually exclusive)** |  |  |  |
| None | 80.0% | 36.4% | 61.5% |
| Mobility | 6.7% | 54.5% | 26.9% |
| Aphasia | 6.7% | 9.1% | 7.7% |
| Cognition | 0% | 0% | 0% |
| Mobility & Aphasia | 6.7% | 0% | 3.8% |
| Mobility & Cognition | 0% | 0% | 0% |
| Aphasia & Cognition | 0% | 0% | 0% |
| Mobility & Aphasia & Cognition | 0% | 0% | 0% |

Mobility impairment defined as moderate or severe problems in walking about or unable to walk about on the Eq5D-5L Mobility item.

Aphasia impairment defined as a score of 3 out of 4 or less on the OCS picture naming task (based on the 5th centile of normative data in the OCS user manual indicating impairment on expressive language).

Cognitive impairment defined as a score of 4 out of 13 or less on the OCS executive mixed task (based on the 5th centile of normative data in the OCS user manual indicating impairment on Task switching/Attention).

1. [↑](#footnote-ref-1)
2. [↑](#footnote-ref-2)
3. [↑](#footnote-ref-3)
