# Supplementary material for: The RETurn to work After stroKE (RETAKE) trial: Findings from a mixed-methods process evaluation of the Early Stroke Specialist Vocational Rehabilitation (ESSVR) intervention
Source: PLoS One. 2024 Oct 9;19(10):e0311101. doi: 10.1371/journal.pone.0311101 (PMC11463838; doi:10.1371/journal.pone.0311101)
Supplement: Supplementary file 10 [file pone.0311101.s006.docx]

S5 Table: Intervention Fidelity Checklist-Occupational Therapists

Fidelity Checklist | **Participant ID:** | **Researcher Initials:**

Stage One: Early Recovery

Component Number	Core/Desirable	Component Description	Evidence of component? Y/N/NA	Data Extraction			Data Source
1.1	Core	RETAKE OT intervenes within 8 weeks of stroke	SELECT	Date of stroke dd/mm/yyyy<br>Date of first contact dd/mm/yyyy	OT must have interevened by:<br>#VALUE!	Moderating factors (if not within 8 weeks):	SELECT
1.2	Core	RETAKE OT assesses the impact of the stroke on the participant	SELECT				SELECT
1.3	Core	RETAKE OT assesses the impact of the stroke on the participant's job	SELECT				SELECT
1.4	Desirable	RETAKE OT assesses the impact of the stroke on the participant's family	SELECT				SELECT
1.5	Core	RETAKE OT helps the participant plan a return to work and prepares them to return to work	SELECT				SELECT
1.6	Core	RETAKE OT communicates in writing with relevant stakeholders regarding work status	SELECT				SELECT
1.7	Core	RETAKE OT coordinates VR across relevant sectors	SELECT				SELECT

Stage Two: Graded Return to Work

Component Number	Core/Desirable	Component Description	Evidence of component?	Data Extraction	Data Source
2.1	Core	RETAKE OT provides education and advice to the participant	SELECT		SELECT
2.2	Desirable	RETAKE OT provides emotional support to the participant	SELECT		SELECT
2.3	Core	RETAKE OT provides education and advice to the employer	SELECT		SELECT
2.4	Desirable	RETAKE OT provides emotional support to the employer	SELECT		SELECT
2.5	Desirable	RETAKE OT provides education and advice to the participant's family	SELECT		SELECT
2.6	Desirable	RETAKE OT provides emotional support to the participant's family	SELECT		SELECT
2.7	Core	RETAKE OT negotiates a phased return to work	SELECT		SELECT
2.8	Core	RETAKE OT mediates workplace adjustments	SELECT		SELECT
2.9	Core	RETAKE OT provides mechanism for feedback based on work performance	SELECT		SELECT
2.10	Core	RETAKE OT continuously monitors the participant's return to work to ensure sustainability and job retention	SELECT		SELECT

Stage Three: Job Retention

Component Number	Core/Desirable	Component Description	Evidence of component?	Data Extraction	Data Source
3.1	Core	RETAKE OT identifies issues that arise within the return to work process with relevant stakeholders.	SELECT		SELECT
3.2	Core	RETAKE OT addresses the issues that arise within the return to work process with all stakeholders	SELECT		SELECT
3.3	Core	RETAKE OT explores alternative duties and/ or job roles for the participant where current work could not be sustained/ was not feasible	SELECT		SELECT
3.4	Core	RETAKE OT practices gradual, appropriate disengagement from the intervention with the participant	SELECT		SELECT
3.5	Core	RETAKE OT discusses gradual, appropriate disengagement from the intervention with the participant's employer	SELECT		SELECT

Stage Four: Discharge Process

Component Number	Core/Desirable	Component Description	Evidence of component?	Data Extraction	Data Source
4.1	Core	RETAKE OT and participant agree an appropriate timepoint for withdrawing from the intervention	SELECT		SELECT
4.2	Core	RETAKE OT discusses and communicates the mechanism for re-accessing the vocational service or provides information about access to further avenues of support to the participant	SELECT		SELECT
4.3	Core	RETAKE OT discusses and communicates the mechanism for re-accessing the vocational service or provides information about access to further avenues of support to the participant's employer	SELECT		SELECT
4.4	Desirable	RETAKE OT discusses and communicates the mechanism for re-accessing the vocational service or provides information about access to further avenues of support to the participant's family	SELECT		SELECT
4.5	Core	RETAKE OT provides the participant's GP and other relevant health care professionals with a copy of the Discharge Letter	SELECT		SELECT

Stage	Core	%	Desirable	%
Stage One	0	0	0	0
Stage Two	0	0	0	0
Stage Three	0	0	NONE	NONE
Stage Four	0	0	0	0
Total	0	0.0	0	0.0

Time Taken to Complete:
