# Supplementary material for: The RETurn to work After stroKE (RETAKE) trial: Findings from a mixed-methods process evaluation of the Early Stroke Specialist Vocational Rehabilitation (ESSVR) intervention
Source: PLoS One. 2024 Oct 9;19(10):e0311101. doi: 10.1371/journal.pone.0311101 (PMC11463838; doi:10.1371/journal.pone.0311101)
Supplement: Attachment — Submitted filename: Response to reviewers.docx [file pone.0311101.s011.docx]

PONE-D-24-17536

The RETurn to work After stroKE (RETAKE) trial: findings from a mixed-methods process evaluation of the Early Stroke Specialist Vocational Rehabilitation (ESSVR) intervention.

| **Revision requested** | **Responses** |
| --- | --- |
| **Editorial revisions** |  |
| Please ensure that your manuscript meets PLOS ONE's style requirements, including those for file naming. | We have reviewed the author guidance, amended the manuscript accordingly, and revised file names in the resubmission accordingly. |
| Thank you for stating the following financial disclosure:  "This study was funded by the NIHR HTA programme (ref: 15/130/11). The views expressed herein are those of the authors, not necessarily the NIHR, the Department of Health and Social Care or the NHS."  Please state what role the funders took in the study.  If the funders had no role, please state: "The funders had no role in study design, data collection and analysis, decision to publish, or preparation of the manuscript."  If this statement is not correct you must amend it as needed.  Please include this amended Role of Funder statement in your cover letter; we will change the online submission form on your behalf. | We have added the statement: "The funders had no role in study design, data collection and analysis, decision to publish, or preparation of the manuscript."  to our cover letter as suggested. |
| Please expand the acronym “NIHR HTA” (as indicated in your financial disclosure) so that it states the name of your funders in full. This information should be included in your cover letter; we will change the online submission form on your behalf. | We have added the full definition of NIHR HTA to our cover letter as suggested. |
| Please note that in order to use the direct billing option the corresponding author must be affiliated with the chosen institute. Please either amend your manuscript to change the affiliation or corresponding author, or email us at [plosone@plos.org](mailto:plosone@plos.org) with a request to remove this option. | We have changed the corresponding author to: Professor Kathryn Radford.  Professor Radford is employed by the University of Nottingham. |
| Please upload a copy of Figure 1, to which you refer in your text on page 8. If the figure is no longer to be included as part of the submission, please remove all reference to it within the text. | We apologise for this oversight. Figure 1 is an important element of the manuscript and is now included in the submission. |
| Please include captions for your Supporting Information files at the end of your manuscript, and update any in-text citations to match accordingly. Please see our Supporting Information guidelines for more information: | We have provided captions in a list as indicated, these captions now match the in text citations for these files. |
| Please review your reference list to ensure that it is complete and correct. If you have cited papers that have been retracted, please include the rationale for doing so in the manuscript text, or remove these references and replace them with relevant current references. Any changes to the reference list should be mentioned in the rebuttal letter that accompanies your revised manuscript. If you need to cite a retracted article, indicate the article’s retracted status in the References list and also include a citation and full reference for the retraction notice. | We have reviewed the reference list and removed references to papers which are either in development or are awaiting review. These has resulted in the removal of 6 references. We have listed the references we have removed in the cover letter as requested. |
| **Reviewer 1 comments** |  |
| Some minor amendments  -Could the authors include a brief anymore details description of the intervention if possible.  - Supplemental file 1 logic model, could family support be a factor to consider for the person returning to work after stroke?    -Supplemental file 3 refers to policy, what policy is this? | Thank you for your feedback on the manuscript.  We have considered this suggestion and added a small amount of additional text to the introduction. Being mindful of the length of the paper we refer to core components of ESSVR which are listed in the logic model (Supplementary File 1) and the protocol for the RETAKE process evaluation (Ref 22) which provides a detailed description of the intervention using the TIDieR checklist.  Family support is a potentially supporting factor in RTW. The ESSVR intervention seeks to actively engage family members in supporting stroke survivors and recognises the likely support needs of family members too. In the process evaluation we sought to understand the involvement of family members. Work commitments for some family members of case study participants impacted on involvement, but our data suggests family member support was an important factor for many stroke survivors. The logic model we present in the manuscript is the pre-trial version which informed the process evaluation. This is likely to be revised post-publication of the trial results. The revised version will take account of influencing factors identified in the trial and process evaluation findings. The inclusion of family response in the individual outcomes element of the logic model is being considered.  This is one of the moderators identified in the CFIF model and, as intended by the authors Carroll et al (2007), relates to the clarity and specificity guidelines or policy which potentially influences implementation fidelity. In the case of RETAKE we focused on the National Clinical Guideline for Stroke (2019, updated 2023) and the NHS England Stroke Service Model (2021) both of which make specific reference for the kind of RTW support which should be offered to stroke survivors in the post-acute care period. These policy guidelines could have influenced the kind of usual care services that stroke survivors received. |
| **Reviewer 2** |  |
|  | Thank you for your feedback on the manuscript, we found the comments and suggestion most helpful in completing the revisions. |
| There is a detailed methods section (page 6-14 of manuscript). The authors methodically outline each component of the process evaluation, with some results, and then refer to earlier publication for full results. The main suggestion is to reduce the detail in the methods and expand on the detail in the results, so that the results better support the discussion/conclusion about the benefit of the program. This needs to be read as a stand-alone paper. | We accept the comment that there could be reduction in the text detailing the methods and further detail in the results sections. We have revised the methods and results sections to take account of this comment. In these revisions we sought to retain sufficient detail to allow replication of the process evaluation methods employed.  We were somewhat unclear about the reviewer’s comment that we refer to earlier publication for full results. We wish to be clear that there is no single earlier publication reporting the results of the process evaluation. However, we acknowledge that we refer to previously published papers relating to the development of a fidelity checklist (Ref 30) and more detailed reporting on embedding mentorship to support intervention implementation (ref 35), within the process evaluation. We had made reference to additional papers which have been submitted for publication but remain in review at this time. These papers will, i) report the main trial results, ii) explore how the OT competency assessment methodology was developed and evaluated, iii) focus on exploration and comparison of individual case examples from the longitudinal case study group participants, iv) report the health economic evaluation, which was a separate study. As indicated above and in the response to the editor’s comments, we have removed reference to these papers from the manuscripts but identified that they will be published elsewhere. We acknowledge that their prior inclusion in this manuscript was unhelpful and may have implied prior reporting of the findings of the process evaluation. Providing more detailed results for each of these elements (excluding the health economic study) would significantly increase the word count and make the current manuscript unwieldy. Nonetheless, we accept that adding some more detail on our findings, in the core areas of the process evaluation would enhance this manuscript. We have added to the manuscript as indicated in the track changes and clean copies of the revised manuscript.  We agree the process evaluation findings manuscript should stand alone and, with the revisions made in line with reviewers’ comments, we now believe it does so. |
| Introduction: • Client group is established but is there a need to define stroke?  • Line 112 – “ .. but limited stroke specific qualitative evidence informs the delivery of effective VR services.” The lead up to this sentence all makes sense and is building an effective argument / statement about the lack of descriptive literature for RTW interventions, however it is hard to make sense of this particular sentence. | We have added a brief definition of stroke to the beginning of the introduction  We accept this comment. We have inserted new text as follows:  Line 116: There is consistent qualitative evidence of unmet need, poor co-ordination of services and time limited support post-stroke [5,17-18]. The RETurn to work After stroKE (RETAKE) trial was developed to address these gaps in the existing evidence related to the provision of effective VR early after stroke. [19] |
| Methods: Figure 1 Process Evaluation flowchart did not seem available, which is the central diagram | We apologise for this oversight. Figure 1 is an important element of the manuscript and is now included in the submission. |
| PPI involvement: Line 162: “A Public and Patient Involvement group was involved throughout the RETAKE study. Involvement included:…”. To reduce the repeated use of “involve” in this sentence, suggest “A Public and Patient Involvement group was engaged throughout the RETAKE study, to: …”. | We have amended this sentence as suggested. |
| Data collection (figure 1): Line 227 : “We collected data at the …”. To avoid the use of “we”, suggest “Data were collected at the… ” | We have amended the text as suggested. |
| Impact of COVID19 pandemic Line 310 and 341: – again, consider re-wording to avoid the use of “we”. | We have amended the text as suggested. |
| Content dose, intensity and duration of ESSVR (Objective 1a):  Line 454: “Trial data indicate that 97.2% (n=307) of ESSVR participants commenced the intervention, with a mean of 41.9% (SD:23.59) within the first month after their stroke”. This reads that 97.2% commenced ESSVR within 1 month of stroke, so then what does the 41.9% represent? Is this correct ? : “Of the 307 participants that commenced the intervention, 41.9% (SD:23.59) were within one month of stroke”. (suggesting re-wording to be clearer) | We agree that this sentence lacked clarity and have reworded the text as suggested. |
| Intervention delivery: facilitators and barriers:  Line 467: “Interactional and skillset workability in collective action…”. This is not familiar terminology. Will the broader readership know what this means? It seems to have been introduced for the first time at this stage and is not relatable. I can see the authors have referenced NPT constructs, but the reader is required to refer to this firstly, in order to understand the commentary. It is suggested the authors manage this translation from theory and insert the practise-oriented results. This issue aside, the authors have captured some important barriers – continued impairments, blocks to liaising with employers, workplace in-house arrangements etc. | Thank you for this comment. The issue of how NPT is referenced in the text is a challenge in many papers. We have reviewed the two sections in which we refer to NPT (OT Intervention delivery-Barriers and Facilitators and Participants’ experiences). In these sections we have removed reference to individual components associated with NPT’s core constructs (e.g. interactional and skill set workability). In these instances, we have added or removed text, where appropriate, to clarify our explanation of the work of intervention delivery or the experiences of ESSVR participants. |
| Line 511 – “Interactional workability and contextual integration…” – refer to comment above | Text amended, see above. |
| Line 523 – the description of OTs working across different trusts is not relatable to an international audience, and whilst meaningful to the local study participants, may not have value in this publication. | We understand the point being made here. We have added to this section by clarifying that NHS Trusts are healthcare provider organisations and used this form of words as part of the explanation of the problems OTs encountered when working across different healthcare provider organisations. |
| Line 549 – “These staff are not stroke-specialist and may not have lacked the skills…”. Should this be “may have lacked…” | Yes, this was a typographical error. |
| Line 586 - 31/03/2020 – should this date be written out in full? | Now written in full. |
| Line 599: “In the case-study group ESSVR plus UC participants 53% (n= 8/15) had RWT at 12 months, for UC only participants 45% (n=5/11) had RTW at 12 months. For the additional interview participants ESSVR plus UC participants 50% (n=4/8) participants had RTW at 12 months and for UC 60% (n= 6/10) had RTW at 12 months.”  This is difficult to wade through. It is established earlier that ESSVR participants also receive UC, therefore, can they be known as “ESSVR participants” in this section? | We have revised this section to remove the reference to ‘plus UC’. |
| Again in the analysis of participant’s experiences the authors refer to NPT theory, eg: Line 608: “Individual stroke survivors had to make sense of their situation post-stroke and undertake RTW related activity (coherence: individual specification)”. There doesn’t seem value in including the NPT theory correlate in brackets. | Thank you for this comment. We understand the point being made. We have amended the participant experiences section in line with the OT section, retaining broad reference to NPTs constructs, so as to be consistent with our intention to present a theory driven process evaluation. However, in line with your comment we have removed the reference to individual components of NPTs constructs such as individual specification. |
| Discussion: Line 754 – 759 : The discussion presents the RTW outcomes for the study, which is new information introduced at the discussion. This is reported in another publication and does not seem linked to the current report. Suggest removing this. | We accept that this statement introduces new information in the discussion section of the manuscript. Its inclusion here was to enter into some discussion about the possible reasons for the finding of no significant difference between the RTW rates in the trial arms. However, we recognise there is limited opportunity in this manuscript to review these issues. We have now removed the section reporting the headline trial result. |
| Conclusion – states that the intervention was highly valued by all parties. There doesn’t seem to be any evidence of the employer experience, so they should be removed from this list of satisfied parties. | We have removed the reference to employers from the first line of the conclusion. |
